# Supplementary figures and images for: MicrobeTrace: Retooling molecular epidemiology for rapid public health response
Source: PLoS Comput Biol. 2021 Sep 7;17(9):e1009300. doi: 10.1371/journal.pcbi.1009300 (PMC8491948; doi:10.1371/journal.pcbi.1009300)

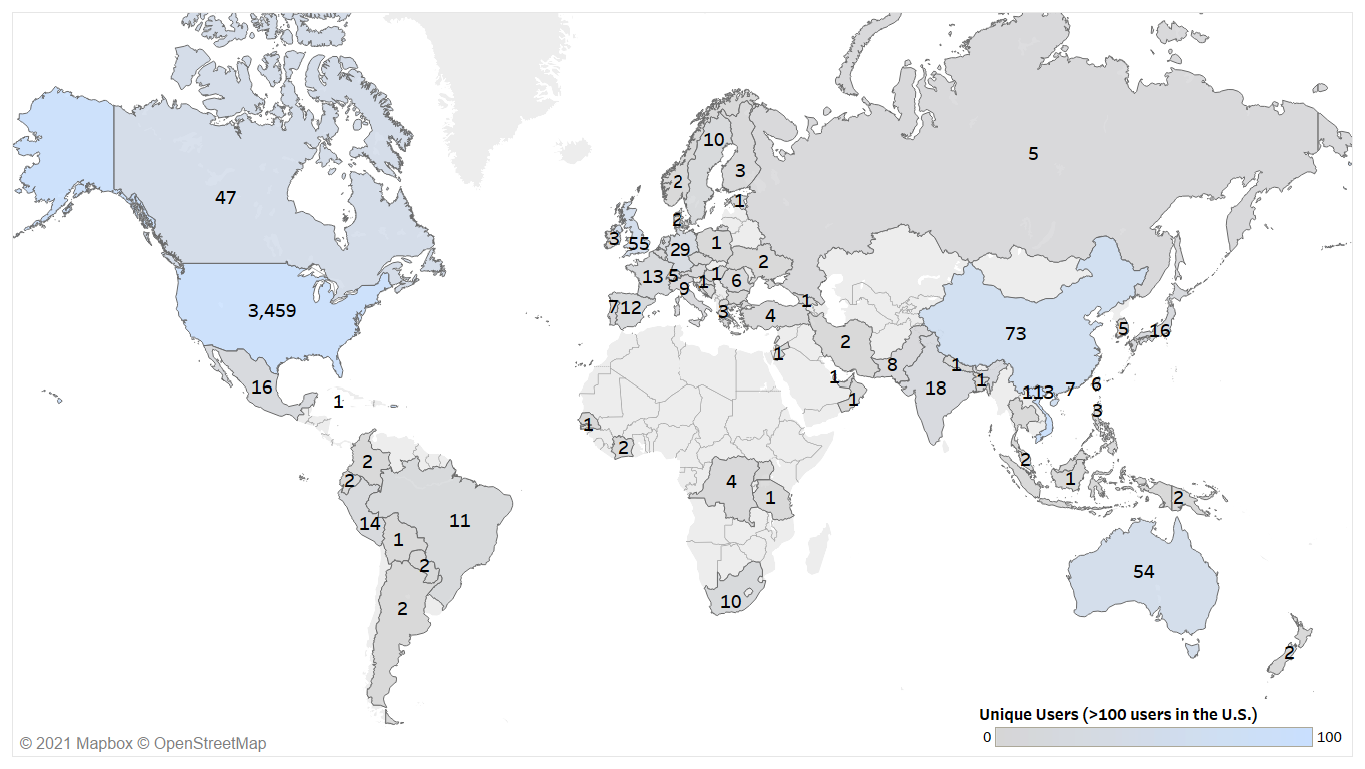

Supplement: S1 Fig — Color shading and marks are labeled by the total number of unique users from March 1st, 2018 to November 30th, 2020. The overwhelming majority of users access MicrobeTrace in the U.S. (84%) and international usage mostly comes from Vietnam (3%), China (2%), United Kingdom (1%), Australia (1%) and Canada (1%). Sixty-five additional countries have <1% users. Maps were created using Tableau.©Mapbox and ©OpenStreetMap are available by default in the Tableau Software® Map Layers pane. Each Tableau Software® map built-in includes acknowledgments of ©Mapbox (https://www.mapbox.com/tableau/) and ©OpenStreetMap (https://www.openstreetmap.org/). ©OpenStreetMap is free to use under an open license. (TIFF) [file pcbi.1009300.s001.tiff]

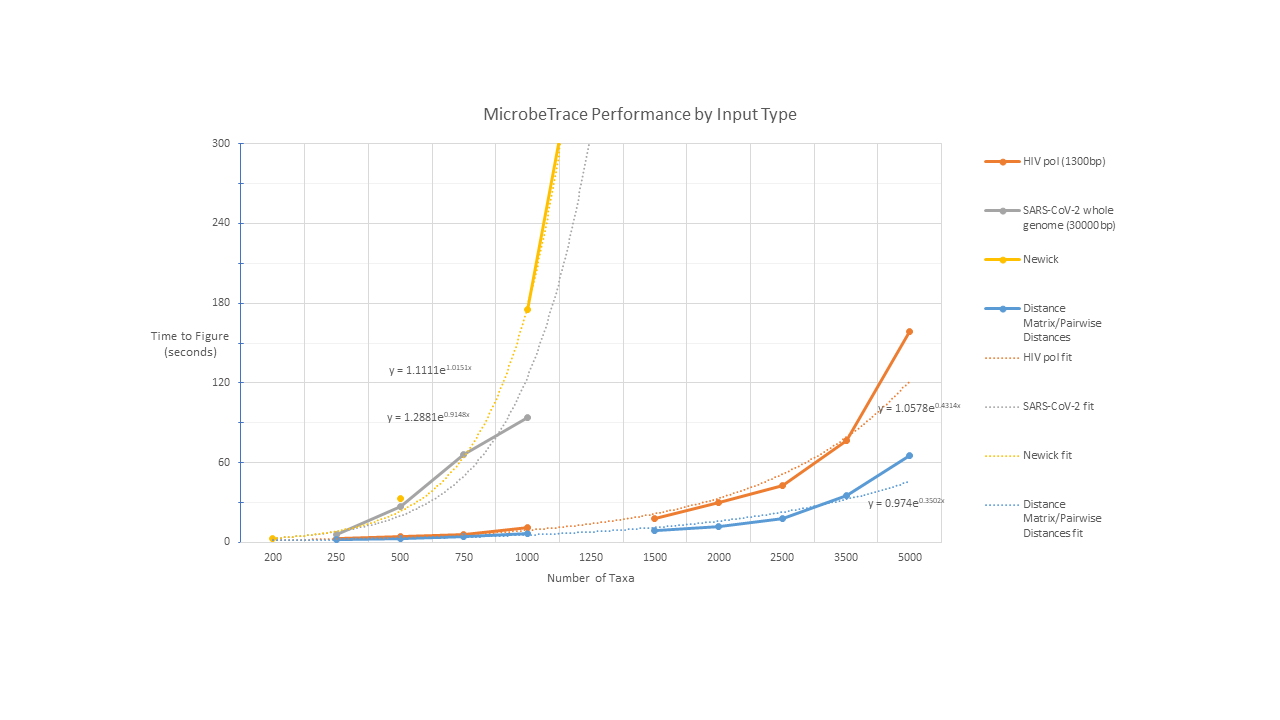

Supplement: S2 Fig — (TIF) [file pcbi.1009300.s002.tif]
