# Supplementary material for: MicrobeTrace: Retooling molecular epidemiology for rapid public health response
Source: PLoS Comput Biol. 2021 Sep 7;17(9):e1009300. doi: 10.1371/journal.pcbi.1009300 (PMC8491948; doi:10.1371/journal.pcbi.1009300)
Supplement: S1 Table — (PDF) [file pcbi.1009300.s003.pdf]

Country	City	Users	New_Users	Sessions	Avg Session Duration (hr)	Time of Use (hr)
United States	Brookhaven	277	145	573	19.214	11009.622
United States	Chamblee	200	180	595	21.932	13049.540
United States	Druid Hills	1,028	563	2,341	91.870	215067.670
United States	Dunwoody	165	24	296	19.871	5881.816
United States	North Druid Hills	6	9	23	1.029	23.667
United States	(not set)	6	68	76	0.486	36.936
United States	Acworth	0	2	2	0.000	0.000
United States	Alamo Heights	2	0	2	0.006	0.012
United States	Albany	7	15	26	0.557	14.482
United States	Albuquerque	0	2	2	0.006	0.012
United States	Alexandria	0	2	2	0.000	0.000
United States	Alhambra	8	6	19	1.770	33.630
United States	Allen	0	1	1	0.000	0.000
United States	Amesbury	19	1	29	2.918	84.622
United States	Anaheim	0	2	3	0.000	0.000
United States	Anchorage	1	6	7	0.031	0.217
United States	Ann Arbor	0	4	4	0.000	0.000
United States	Annandale	2	3	5	0.009	0.045
United States	Ardmore	0	1	1	0.002	0.002
United States	Arlington	1	4	5	0.046	0.230
United States	Arnold	2	0	4	0.265	1.060
United States	Ashburn	22	437	480	3.434	1648.320
United States	Athens	0	2	2	0.089	0.178
United States	Atlanta	532	376	1,514	58.515	88591.710
United States	Auburn	0	1	1	0.048	0.048
United States	Augusta	11	5	21	2.091	43.911
United States	Austin	12	32	52	3.036	157.872
United States	Avon	0	1	1	0.001	0.001
United States	Bainbridge Island	8	3	14	0.900	12.600
United States	Ballwin	0	1	1	0.000	0.000
United States	Baltimore	44	34	118	5.698	672.364
United States	Bar Harbor	1	3	4	0.535	2.140
United States	Barrington	0	1	1	0.134	0.134
United States	Baytown	21	2	37	1.768	65.416
United States	Bedford	0	1	1	0.000	0.000
United States	Bel Air South	0	1	1	0.016	0.016
United States	Bellevue	0	1	1	0.000	0.000
United States	Berkeley	1	2	3	0.002	0.006
United States	Bernardsville	3	1	5	0.071	0.355
United States	Bethesda	2	10	14	0.002	0.028
United States	Beverly	0	1	1	0.000	0.000
United States	Billings	1	2	4	0.010	0.040
United States	Bismarck	5	3	10	0.901	9.010
United States	Blaine	0	1	1	0.001	0.001
United States	Bloomington	0	1	1	0.406	0.406
United States	Blue Bell	0	1	1	0.001	0.001
United States	Boardman	0	44	44	0.000	0.000
United States	Bohemia	0	1	1	0.001	0.001
United States	Boise	2	2	5	0.244	1.220
United States	Boston	112	58	238	13.060	3108.280
United States	Brandon	3	1	10	1.785	17.850
United States	Brandywine	2	2	6	0.008	0.048
United States	Bremerton	0	1	1	0.000	0.000
United States	Brentwood	0	1	1	0.000	0.000
United States	Bristow	0	2	2	0.019	0.038
United States	Broken Bow	1	0	1	0.026	0.026
United States	Brookline	0	3	3	0.000	0.000
United States	Buellton	1	1	2	0.000	0.000
United States	Burke	0	3	3	0.012	0.036
United States	Burlingame	0	1	1	0.001	0.001
United States	Burlington	3	12	20	1.560	31.200
United States	Camas	0	1	1	0.000	0.000

Country	City	Users	New_Users	Sessions	Avg Session Duration (hr)	Time of Use (hr)
United States	Cambridge	19	15	48	2.378	114.144
United States	Campbell	0	1	1	0.000	0.000
United States	Canton	0	1	1	0.026	0.026
United States	Cape Saint Claire	0	1	1	0.000	0.000
United States	Carey	2	0	2	0.008	0.016
United States	Carson City	1	2	3	0.000	0.000
United States	Cary	1	0	1	0.426	0.426
United States	Cedar Park	0	1	1	0.001	0.001
United States	Ceres	0	1	1	0.000	0.000
United States	Chantilly	4	6	11	0.036	0.396
United States	Charleston	2	8	19	0.677	12.863
United States	Charlotte	2	3	5	0.248	1.240
United States	Charlottesville	0	1	1	0.000	0.000
United States	Cheyenne	1	50	51	0.050	2.550
United States	Chicago	7	37	46	0.468	21.528
United States	Chillum	1	0	1	0.000	0.000
United States	Christiansburg	0	1	1	0.006	0.006
United States	Cincinnati	1	7	9	0.253	2.277
United States	Clarksburg	0	1	1	0.000	0.000
United States	Clarksville	7	7	20	0.194	3.880
United States	Clayton	0	1	1	0.000	0.000
United States	Cocoa Beach	0	1	1	0.000	0.000
United States	Coffeyville	0	8	8	0.000	0.000
United States	Colchester	7	3	12	0.014	0.168
United States	Cold Spring Harbor	0	1	1	0.000	0.000
United States	College Park	2	0	2	0.004	0.008
United States	Collierville	0	1	1	0.000	0.000
United States	Colorado Springs	5	2	17	0.492	8.364
United States	Columbia	4	8	13	0.288	3.744
United States	Columbus	36	23	83	7.054	585.482
United States	Concord	1	1	2	0.008	0.016
United States	Corpus Christi	0	2	2	0.476	0.952
United States	Covington	0	1	1	0.001	0.001
United States	Culver City	1	0	1	0.000	0.000
United States	Cumming	0	2	2	1.148	2.296
United States	Cupertino	0	1	1	0.001	0.001
United States	Cypress	0	2	2	0.000	0.000
United States	Dallas	0	6	7	0.080	0.560
United States	Danville	0	1	2	0.026	0.052
United States	Davis	0	2	2	0.498	0.996
United States	Decatur	13	21	42	2.684	112.728
United States	Delhi	0	1	1	0.000	0.000
United States	Delmar	1	0	1	0.000	0.000
United States	Delta charter Township	1	1	2	0.000	0.000
United States	Denton	0	1	1	0.000	0.000
United States	Des Moines	1	11	12	0.067	0.804
United States	Dover	17	11	35	3.132	109.620
United States	Draper	0	1	1	0.000	0.000
United States	Duluth	9	9	22	0.896	19.712
United States	Durham	2	7	9	1.229	11.061
United States	Eagan	0	2	2	0.000	0.000
United States	East Bridgewater	0	1	1	0.000	0.000
United States	East Lansing	2	4	8	0.088	0.704
United States	El Dorado Hills	0	1	2	0.646	1.292
United States	El Reno	0	1	1	0.000	0.000
United States	Elkins	1	0	1	0.000	0.000
United States	Ellicott City	0	1	1	0.000	0.000
United States	Emeryville	1	0	1	0.000	0.000
United States	Evansville	0	1	1	0.000	0.000
United States	Everett	9	0	20	1.534	30.680
United States	Federal Way	0	1	1	0.012	0.012
United States	Flagstaff	6	4	12	0.845	10.140
United States	Florence	2	4	7	0.934	6.538
United States	Forrest City	0	2	2	0.000	0.000

Country	City	Users	New_Users	Sessions	Avg Session Duration (hr)	Time of Use (hr)
United States	Fort Belvoir	1	6	8	0.117	0.936
United States	Fort Collins	0	6	6	0.012	0.072
United States	Fort Mitchell	0	1	1	0.001	0.001
United States	Foster City	1	2	3	0.005	0.015
United States	Frankfort	28	21	61	4.059	247.599
United States	Franklin	0	2	2	0.303	0.606
United States	Franklin Lakes	2	2	5	0.130	0.650
United States	Freeport	0	1	1	0.000	0.000
United States	Fremont	0	1	1	0.001	0.001
United States	Fulton	0	1	1	0.000	0.000
United States	Gainesville	67	28	123	6.148	756.204
United States	Gaithersburg	0	2	2	0.013	0.026
United States	Garden Grove	0	1	2	0.000	0.000
United States	Georgetown	0	1	1	0.001	0.001
United States	Germantown	0	4	4	0.377	1.508
United States	Gillette	0	1	1	0.000	0.000
United States	Glendale	1	5	6	0.251	1.506
United States	Glenside	0	1	1	0.044	0.044
United States	Goose Creek	0	1	1	0.000	0.000
United States	Grafton	1	0	2	0.000	0.000
United States	Granby	0	1	1	0.000	0.000
United States	Greensburg	1	2	3	0.002	0.006
United States	Greenville	2	0	8	0.323	2.584
United States	Grovetown	0	1	1	0.000	0.000
United States	Hamshire	0	1	1	0.003	0.003
United States	Harpswell	1	0	1	0.001	0.001
United States	Harrison	0	3	3	0.000	0.000
United States	Hartford	2	4	6	0.391	2.346
United States	Havre de Grace	0	2	2	0.000	0.000
United States	Helena	0	4	4	0.287	1.148
United States	Hialeah Gardens	0	2	2	0.000	0.000
United States	Highlands Ranch	6	2	13	0.231	3.003
United States	Holladay	0	1	1	0.000	0.000
United States	Honolulu	0	6	6	0.001	0.006
United States	Houston	31	16	66	3.033	200.178
United States	Huntington	3	0	8	1.069	8.552
United States	Huntsville	0	3	4	0.088	0.352
United States	Hyattsville	0	4	4	0.000	0.000
United States	Ijamsville	1	0	1	0.000	0.000
United States	Indianapolis	2	4	7	0.001	0.007
United States	Iowa City	1	5	7	0.001	0.007
United States	Irving	0	1	1	0.004	0.004
United States	Isla Vista	0	1	1	0.000	0.000
United States	Ithaca	5	3	17	0.680	11.560
United States	Jackson	1	1	3	0.033	0.099
United States	Jefferson City	2	0	2	0.000	0.000
United States	Johns Creek	4	3	7	0.018	0.126
United States	Joplin	0	1	1	0.002	0.002
United States	Kendall West	0	1	1	0.000	0.000
United States	Kent	0	1	1	0.000	0.000
United States	Kingston	2	2	4	0.072	0.288
United States	Kirkwood	0	1	1	0.000	0.000
United States	Kutztown	1	3	5	0.562	2.810
United States	La Crescenta-Montrose	0	1	1	0.000	0.000
United States	Lake Butler	0	1	1	0.000	0.000
United States	Lake Station	0	2	2	0.000	0.000
United States	Lakeside	7	4	12	0.012	0.144
United States	Lakewood	0	1	1	0.000	0.000
United States	Lansing	13	19	41	2.446	100.286
United States	Las Cruces	0	2	2	0.134	0.268
United States	Latham	0	1	1	0.000	0.000
United States	Laurel	0	1	1	0.006	0.006
United States	Laurel Hollow	0	1	1	0.000	0.000
United States	Lawrenceville	0	2	2	0.000	0.000

Country	City	Users	New_Users	Sessions	Avg Session Duration (hr)	Time of Use (hr)
United States	League City	0	1	1	0.000	0.000
United States	Lebanon	0	1	1	0.008	0.008
United States	Lenexa	0	2	5	0.103	0.515
United States	Lexington	0	1	1	0.000	0.000
United States	Lilburn	0	1	1	0.002	0.002
United States	Little Rock	43	14	95	5.389	511.955
United States	Lochearn	1	1	2	0.002	0.004
United States	Loganville	0	1	1	0.000	0.000
United States	Los Alamos	4	4	8	0.228	1.824
United States	Los Angeles	17	24	50	1.312	65.600
United States	Louisville	0	4	7	0.020	0.140
United States	Madison	3	6	10	0.191	1.910
United States	Malden	3	1	5	0.217	1.085
United States	Manchester	1	1	2	0.002	0.004
United States	Mansfield	0	2	3	0.004	0.012
United States	Marietta	1	0	3	0.370	1.110
United States	Mebane	0	1	1	0.000	0.000
United States	Medford	1	1	2	0.005	0.010
United States	Memphis	1	0	1	0.054	0.054
United States	Menands	0	1	1	0.000	0.000
United States	Menlo Park	0	1	1	0.000	0.000
United States	Meridian charter Township	0	1	1	0.000	0.000
United States	Mesa	1	1	3	0.000	0.000
United States	Miami	3	4	8	0.000	0.000
United States	Miami Beach	1	0	1	0.000	0.000
United States	Millcreek	1	0	1	0.083	0.083
United States	Milledgeville	0	1	1	0.000	0.000
United States	Milton	1	3	4	0.553	2.212
United States	Minneapolis	3	2	5	0.009	0.045
United States	Minnetonka	1	0	1	0.000	0.000
United States	Mint Hill	0	1	1	0.000	0.000
United States	Missouri City	0	3	3	0.019	0.057
United States	Montgomery	2	0	2	0.023	0.046
United States	Montpelier	17	43	64	1.118	71.552
United States	Moorpark	0	2	2	0.000	0.000
United States	Morgantown	0	1	1	0.001	0.001
United States	Moses Lake	0	1	1	0.001	0.001
United States	Mountain Park	4	6	11	0.600	6.600
United States	Mountain View	0	1	1	0.002	0.002
United States	Nashville	58	18	94	4.640	436.160
United States	New Haven	0	4	4	0.001	0.004
United States	New London	0	1	1	0.000	0.000
United States	New Orleans	0	3	3	0.003	0.009
United States	New York	9	57	77	1.118	86.086
United States	Newport Beach	0	1	1	0.000	0.000
United States	Norfolk	0	1	1	0.021	0.021
United States	North Bethesda	3	5	11	0.227	2.497
United States	North Creek	0	1	1	0.001	0.001
United States	North Decatur	66	28	147	7.102	1043.994
United States	North Little Rock	4	0	4	1.130	4.520
United States	Northampton	0	1	1	0.000	0.000
United States	Oak Lawn	0	1	1	0.000	0.000
United States	Oak Park	0	1	1	0.000	0.000
United States	Oak Ridge	0	1	1	0.014	0.014
United States	Oakland	1	8	11	0.107	1.177
United States	Oakton	0	2	2	0.000	0.000
United States	Oceanside	1	1	2	0.646	1.292
United States	Oklahoma City	0	2	2	0.241	0.482
United States	Olney	1	0	1	0.000	0.000
United States	Olympia	35	5	92	4.774	439.208
United States	Omaha	0	1	1	0.000	0.000
United States	Ontario	0	1	1	0.000	0.000
United States	Orlando	1	2	3	0.018	0.054
United States	Palo Alto	1	4	6	0.008	0.048

Country	City	Users	New_Users	Sessions	Avg Session Duration (hr)	Time of Use (hr)
United States	Patterson	0	1	1	0.000	0.000
United States	Peachtree City	0	2	2	0.002	0.004
United States	Pearl River	0	1	1	0.001	0.001
United States	Pepper Pike	0	1	1	0.000	0.000
United States	Philadelphia	16	17	39	2.321	90.519
United States	Phoenix	17	20	45	2.750	123.750
United States	Pierre	0	1	1	0.004	0.004
United States	Pittsburgh	1	0	1	0.000	0.000
United States	Placitas	0	1	1	0.000	0.000
United States	Pleasanton	0	1	1	0.197	0.197
United States	Pompano Beach	0	1	1	0.000	0.000
United States	Portland	6	7	20	0.327	6.540
United States	Potomac	0	1	1	0.001	0.001
United States	Providence	8	7	21	0.452	9.492
United States	Pullman	0	1	1	0.000	0.000
United States	Quincy	0	42	43	0.016	0.688
United States	Raleigh	4	6	14	0.309	4.326
United States	Redmond	0	1	1	0.000	0.000
United States	Redwood City	0	1	1	0.007	0.007
United States	Reno	1	0	1	0.000	0.000
United States	Reston	21	17	44	4.605	202.620
United States	Richardson	0	1	1	0.002	0.002
United States	Richmond	1	6	7	0.544	3.808
United States	Riverside	0	1	1	0.000	0.000
United States	Rockledge	0	1	1	0.000	0.000
United States	Rockville	3	3	6	0.388	2.328
United States	Rockwood	0	2	2	0.000	0.000
United States	Rocky Hill	1	7	8	0.000	0.000
United States	Rogers	5	0	7	1.782	12.474
United States	Roswell	0	5	8	0.085	0.680
United States	Round Rock	1	3	4	0.018	0.072
United States	Sacramento	46	40	101	3.289	332.189
United States	Saint Paul	22	29	63	2.248	141.624
United States	Salt Lake City	15	4	27	0.693	18.711
United States	San Antonio	0	6	9	0.334	3.006
United States	San Bernardino	0	2	2	0.001	0.002
United States	San Clemente	1	1	2	0.203	0.406
United States	San Diego	14	32	53	0.986	52.258
United States	San Dimas	0	2	2	0.038	0.076
United States	San Francisco	4	18	22	0.017	0.374
United States	San Jose	1	1	2	0.001	0.002
United States	San Marino	0	1	2	0.067	0.134
United States	San Rafael	0	1	1	0.000	0.000
United States	Sandy Springs	90	10	211	15.557	3282.527
United States	Santa Ana	0	2	2	0.000	0.000
United States	Santa Clara	0	8	8	0.001	0.008
United States	Santa Cruz	1	1	2	0.005	0.010
United States	Santa Fe	0	2	2	0.036	0.072
United States	Sartell	4	0	8	0.262	2.096
United States	Scottdale	1	4	5	0.013	0.065
United States	Seattle	36	29	83	3.601	298.883
United States	Seward	0	1	1	0.001	0.001
United States	Shaker Heights	0	1	1	0.005	0.005
United States	Shoreline	37	14	62	3.810	236.220
United States	Shoreview	1	0	1	0.134	0.134
United States	Silver Spring	2	6	9	0.198	1.782
United States	Simpsonville	1	2	3	0.819	2.457
United States	Sioux Falls	0	1	1	0.000	0.000
United States	Snellville	1	3	4	0.000	0.000
United States	Somerville	0	2	2	0.020	0.040
United States	South San Francisco	0	1	1	0.000	0.000
United States	Southfield	1	1	2	0.000	0.000
United States	Springfield	3	2	6	0.579	3.474
United States	St. Louis	0	1	1	0.000	0.000

Country	City	Users	New_Users	Sessions	Avg Session Duration (hr)	Time of Use (hr)
United States	Stafford	0	1	2	0.000	0.000
United States	Stoughton	3	2	6	0.058	0.348
United States	Sugar Land	34	5	67	3.098	207.566
United States	Summerville	0	1	1	0.000	0.000
United States	Sun Prairie	0	1	1	0.000	0.000
United States	Sunnyvale	0	1	1	0.000	0.000
United States	Surf City	1	0	1	0.001	0.001
United States	Suwanee	0	2	2	0.000	0.000
United States	Tallahassee	21	16	44	2.246	98.824
United States	Tamarac	0	1	1	0.007	0.007
United States	Tampa	4	3	8	1.300	10.400
United States	Teays Valley	0	1	1	0.000	0.000
United States	The Villages	0	1	1	0.007	0.007
United States	Toledo	0	2	2	0.071	0.142
United States	Topeka	0	1	1	0.000	0.000
United States	Troy	0	1	1	0.000	0.000
United States	Tucker	23	11	43	4.858	208.894
United States	Tumwater	67	8	133	7.150	950.950
United States	Tysons	0	3	3	0.000	0.000
United States	Union Park	6	7	17	0.914	15.538
United States	Verona	1	1	3	0.028	0.084
United States	Voorheesville	0	1	1	0.013	0.013
United States	Wallingford	0	1	1	0.000	0.000
United States	Walnut Creek	1	0	1	0.299	0.299
United States	Waltham	1	3	4	0.040	0.160
United States	Washington	6	98	110	0.807	88.770
United States	Waterloo	0	1	1	0.001	0.001
United States	Waukegan	1	0	1	0.229	0.229
United States	Weatherford	0	1	1	0.000	0.000
United States	West Lafayette	0	1	1	0.000	0.000
United States	West Valley City	4	3	8	0.104	0.832
United States	Westminster	0	1	1	0.000	0.000
United States	Wildwood	0	1	1	0.000	0.000
United States	Wilmington Island	0	1	1	0.000	0.000
United States	Winchester	0	2	2	0.001	0.002
United States	Winder	0	1	1	0.000	0.000
United States	Windham	0	1	1	0.000	0.000
United States	Windsor	1	0	1	0.000	0.000
United States	Winthrop Harbor	0	1	1	0.000	0.000
United States	Woodbury	1	1	3	0.028	0.084
United States	Woodlawn	0	4	5	0.021	0.105
United States	Woodstock	1	1	2	0.011	0.022
United States	Worcester	0	1	1	0.242	0.242
United States	Wynnewood	0	1	1	0.000	0.000
United States	Wyomissing	0	2	2	0.000	0.000
United States	Zionsville	2	1	4	1.050	4.200
Vietnam	(not set)	0	5	6	0.000	0.000
Vietnam	Buon Ma Thuot	3	6	11	0.706	7.766
Vietnam	Ca Mau	0	1	1	0.064	0.064
Vietnam	Can Tho	1	0	1	0.000	0.000
Vietnam	Da Nang	10	1	28	3.075	86.100
Vietnam	Dalat	0	1	1	0.455	0.455
Vietnam	Djong Ha	0	2	4	0.649	2.596
Vietnam	Hai Phong	2	1	4	1.925	7.700
Vietnam	Hanoi	80	66	219	10.880	2382.720
Vietnam	Ho Chi Minh City	9	10	28	1.731	48.468
Vietnam	Hoang Mai	0	1	2	0.387	0.774
Vietnam	Hue	0	1	1	0.024	0.024
Vietnam	Nam Djinh	0	1	1	0.000	0.000
Vietnam	Nha Trang	1	2	4	0.000	0.000
Vietnam	Phuc Yen	0	2	2	0.002	0.004
Vietnam	Son Tay	0	2	2	0.346	0.692
Vietnam	Tam Djiep	0	1	1	0.000	0.000
Vietnam	Tam Ky	1	3	4	0.422	1.688

Country	City	Users	New_Users	Sessions	Avg Session Duration (hr)	Time of Use (hr)
Vietnam	Thanh Hoa	0	1	1	0.265	0.265
Vietnam	Tu Son	3	5	11	0.637	7.007
Vietnam	Vinh	0	1	1	0.001	0.001
China	(not set)	0	2	2	0.000	0.000
China	Beijing	0	9	9	0.196	1.764
China	Chengdu	0	1	1	0.000	0.000
China	Chongqing	0	1	1	0.000	0.000
China	Fuzhou	0	5	5	0.000	0.000
China	Guangzhou	0	3	3	0.050	0.150
China	Hangzhou	0	4	4	0.155	0.620
China	Hefei	0	1	1	0.000	0.000
China	Kunming	0	1	1	0.000	0.000
China	Lhasa	0	1	1	0.000	0.000
China	Nanjing	0	3	3	0.000	0.000
China	Nanning	0	1	1	0.000	0.000
China	Nanping	0	1	1	0.000	0.000
China	Ningde	0	3	3	0.000	0.000
China	Putian	0	16	16	0.001	0.016
China	Sanming	0	3	3	0.000	0.000
China	Shanghai	0	2	2	0.000	0.000
China	Shenyang	0	1	1	0.000	0.000
China	Shijiazhuang	0	1	1	0.000	0.000
China	Tianshui	0	1	1	0.000	0.000
China	Urumqi	0	5	5	0.000	0.000
China	Wuhan	0	2	2	0.000	0.000
China	Wuxi	0	1	1	0.000	0.000
China	Yinchuan	0	1	1	0.000	0.000
China	Zhangzhou	0	1	1	0.000	0.000
China	Zhengzhou	0	3	3	0.000	0.000
United Kingdom	(not set)	1	6	8	0.000	0.000
United Kingdom	Cambridge	0	3	3	0.027	0.081
United Kingdom	Edinburgh	0	1	1	0.025	0.025
United Kingdom	Glasgow	4	6	12	1.733	20.796
United Kingdom	Haverhill	0	1	1	0.002	0.002
United Kingdom	Hemel Hempstead	0	1	1	0.000	0.000
United Kingdom	Ipswich	0	1	1	0.000	0.000
United Kingdom	Kidderminster	0	1	1	0.000	0.000
United Kingdom	Leatherhead	0	1	1	0.008	0.008
United Kingdom	Liverpool	0	1	1	0.012	0.012
United Kingdom	London	15	19	41	2.037	83.517
United Kingdom	Loughborough	0	2	2	0.265	0.530
United Kingdom	Manchester	0	1	1	0.000	0.000
United Kingdom	North Shields	0	1	1	0.000	0.000
United Kingdom	Nottingham	1	1	2	0.000	0.000
United Kingdom	Oxford	0	2	2	0.001	0.002
United Kingdom	Reading	1	2	5	0.032	0.160
United Kingdom	Redditch	0	1	1	0.000	0.000
United Kingdom	Slough	0	1	1	0.000	0.000
United Kingdom	Stoke-on-Trent	0	1	1	0.003	0.003
United Kingdom	Trowbridge	0	1	1	0.000	0.000
United Kingdom	Wokingham	3	1	4	0.086	0.344
Australia	(not set)	0	2	2	0.007	0.014
Australia	Brisbane	0	4	4	0.722	2.888
Australia	Canberra	0	1	1	0.013	0.013
Australia	Melbourne	19	24	57	9.445	538.365
Australia	Moe - Newborough	0	1	1	0.000	0.000
Australia	Newcastle	2	6	10	0.371	3.710
Australia	Perth	0	1	2	0.013	0.026
Australia	Sydney	4	15	22	1.698	37.356
Canada	(not set)	0	1	1	0.000	0.000
Canada	Belleville	0	1	1	0.005	0.005
Canada	Brampton	0	1	1	0.000	0.000
Canada	Calgary	0	2	2	0.042	0.084
Canada	Edmonton	0	6	8	0.015	0.120

Country	City	Users	New_Users	Sessions	Avg Session Duration (hr)	Time of Use (hr)
Canada	Guelph	0	1	1	0.000	0.000
Canada	Jasper	1	0	1	0.064	0.064
Canada	Kitchener	0	1	1	0.000	0.000
Canada	Laval	0	1	1	0.000	0.000
Canada	London	0	1	1	0.159	0.159
Canada	Milton	0	2	2	0.074	0.148
Canada	Mississauga	0	1	1	0.001	0.001
Canada	Montreal	2	4	16	0.678	10.848
Canada	Oakville	0	1	1	0.014	0.014
Canada	Ottawa	4	3	8	0.038	0.304
Canada	Red Deer	0	1	1	0.000	0.000
Canada	Richmond	0	1	1	0.001	0.001
Canada	Saint-Hyacinthe	0	2	2	0.001	0.002
Canada	Saskatoon	0	1	1	0.054	0.054
Canada	Thompson	1	1	2	0.006	0.012
Canada	Toronto	1	6	10	0.140	1.400
Canada	Vancouver	0	4	6	0.005	0.030
Canada	Vaughan	0	1	1	0.001	0.001
Canada	Whitehorse	7	2	10	0.543	5.430
Canada	Winnipeg	6	2	17	1.422	24.174
Argentina	Cordoba	0	1	1	0.019	0.019
Argentina	Tandil	0	1	2	0.002	0.004
Austria	Vienna	0	5	5	0.034	0.170
Bangladesh	Dhaka	0	1	1	0.005	0.005
Belgium	Antwerp	0	1	1	0.000	0.000
Belgium	Hasselt	0	1	1	0.000	0.000
Belgium	Ixelles	0	1	1	0.000	0.000
Belgium	Leuven	0	2	2	0.845	1.690
Belgium	Temse	0	1	1	0.008	0.008
Brazil	(not set)	0	2	2	0.014	0.028
Brazil	Brasilia	0	1	2	0.000	0.000
Brazil	Campinas	0	1	2	0.011	0.022
Brazil	Porto Alegre	0	1	1	0.010	0.010
Brazil	Santa Barbara d'Oeste	0	1	1	0.000	0.000
Brazil	Sao Jose do Rio Preto	0	2	2	0.000	0.000
Brazil	Sao Paulo	0	2	2	0.000	0.000
Brazil	Taquaritinga	0	1	1	0.000	0.000
Bulgaria	Sofia	3	2	5	0.269	1.345
Cayman Islands	George Town	0	1	1	0.000	0.000
Colombia	Bogota	0	1	1	0.000	0.000
Colombia	Cali	1	1	2	0.000	0.000
Congo - Kinshasa	Goma	1	4	5	0.175	0.875
Croatia	Zagreb	0	1	1	0.000	0.000
Czechia	Brno	0	1	1	0.002	0.002
Czechia	Hradec Kralove	0	1	1	0.002	0.002
Czechia	Prague	0	2	2	0.027	0.054
Denmark	Fredericia	0	1	1	0.001	0.001
Denmark	Karlslunde	0	1	2	0.000	0.000
Estonia	Tallinn	0	1	1	0.000	0.000
Finland	Espoo	0	1	1	0.001	0.001
Finland	Helsinki	0	2	2	0.000	0.000
France	(not set)	0	3	3	0.003	0.009
France	Betton	0	1	1	0.004	0.004
France	Brest	0	1	1	0.000	0.000
France	Clermont-Ferrand	0	1	1	0.336	0.336
France	Maisons-Alfort	0	1	1	0.000	0.000
France	Montpellier	0	1	1	0.009	0.009
France	Paris	0	4	4	0.005	0.020
France	Tours	0	1	1	0.000	0.000
Gambia	Serrekunda	0	1	1	0.000	0.000
Georgia	Tbilisi	1	1	4	0.629	2.516
Germany	(not set)	0	1	1	0.000	0.000
Germany	Berlin	8	16	31	5.471	169.601
Germany	Cologne	0	1	1	0.000	0.000

Country	City	Users	New_Users	Sessions	Avg Session Duration (hr)	Time of Use (hr)
Germany	Frankfurt	0	1	1	0.000	0.000
Germany	Freiburg im Breisgau	0	1	1	0.000	0.000
Germany	Hamburg	3	3	8	1.480	11.840
Germany	Munich	0	1	1	0.000	0.000
Germany	Nuremberg	0	1	1	0.005	0.005
Germany	Saarbrucken	0	3	3	0.002	0.006
Germany	Stuttgart	0	1	1	0.250	0.250
Greece	Athens	0	1	1	0.000	0.000
Greece	Thessaloniki	0	2	3	0.001	0.003
Hungary	Szeged	0	1	1	0.009	0.009
India	Bengaluru	0	6	6	0.010	0.060
India	Chennai	0	2	2	0.000	0.000
India	Delhi	0	2	2	0.000	0.000
India	Ghaziabad	0	1	1	0.069	0.069
India	Hyderabad	1	1	3	0.028	0.084
India	Jalgaon	0	1	1	0.000	0.000
India	Kochi	0	1	1	0.004	0.004
India	Kolkata	0	1	1	0.108	0.108
India	Mumbai	0	1	1	0.022	0.022
India	Noida	0	1	1	0.002	0.002
India	Viluppuram	0	1	1	0.000	0.000
Indonesia	Denpasar	0	1	1	0.000	0.000
Ireland	Dublin	0	1	1	0.000	0.000
Ireland	Greystones	0	1	1	0.000	0.000
Ireland	Naas	0	1	1	0.032	0.032
Italy	(not set)	0	1	1	0.004	0.004
Italy	Bari	0	1	1	0.000	0.000
Italy	Bolzano	0	1	1	0.004	0.004
Italy	Milan	0	2	2	0.065	0.130
Italy	Rome	0	4	4	0.007	0.028
Japan	Hadano	0	1	2	0.000	0.000
Japan	Hino	0	1	1	0.005	0.005
Japan	Kita City	0	1	1	0.096	0.096
Japan	Kiyose	0	1	1	0.001	0.001
Japan	Matsuyama	0	2	2	0.031	0.062
Japan	Minato City	0	2	2	0.006	0.012
Japan	Okinawa	0	1	1	0.011	0.011
Japan	Oyama	0	5	5	0.000	0.000
Japan	Sapporo	0	1	1	0.000	0.000
Japan	Shibuya City	0	1	1	0.000	0.000
Malaysia	Johor Bahru	2	1	5	0.253	1.265
Malaysia	Kuala Lumpur	1	0	1	0.068	0.068
Malaysia	Petaling Jaya	4	0	5	0.658	3.290
Malaysia	Shah Alam	9	1	27	2.392	64.584
Mexico	(not set)	4	2	13	0.689	8.957
Mexico	Cancun	0	1	1	0.008	0.008
Mexico	Cuernavaca	2	0	2	0.000	0.000
Mexico	Culiacan	8	4	18	1.754	31.572
Mexico	Mazatlan	0	1	1	0.000	0.000
Mexico	Merida	0	1	1	0.022	0.022
Mexico	Mexico City	2	4	9	0.454	4.086
Mexico	Monterrey	0	1	1	0.063	0.063
Mexico	Tijuana	0	2	2	0.075	0.150
Nepal	Kathmandu	0	1	1	0.000	0.000
Netherlands	(not set)	0	1	1	0.004	0.004
Netherlands	Amstelveen	0	1	1	0.001	0.001
Netherlands	Amsterdam	0	3	3	0.000	0.000
Netherlands	Heerlen	0	1	1	0.000	0.000
Netherlands	Rotterdam	10	8	24	1.120	26.880
Netherlands	Utrecht	1	2	4	0.010	0.040
Netherlands	Veenendaal	0	1	1	0.000	0.000
New Zealand	Auckland	0	1	1	0.000	0.000
New Zealand	Wellington	0	1	1	0.000	0.000
North Macedonia	(not set)	0	1	1	0.000	0.000

Country	City	Users	New_Users	Sessions	Avg Session Duration (hr)	Time of Use (hr)
North Macedonia	Skopje	0	1	1	0.000	0.000
Norway	Baerum	0	1	2	0.001	0.002
Norway	Tromso Municipality	0	1	1	0.309	0.309
Pakistan	Lahore	0	8	8	0.000	0.000
Papua New Guinea	Port Moresby	0	2	2	0.023	0.046
Paraguay	Asuncion	0	2	2	0.000	0.000
Philippines	Calamba	0	1	1	0.313	0.313
Philippines	Cebu City	0	1	1	0.001	0.001
Philippines	Manila	1	0	2	0.139	0.278
Philippines	Paranaque	0	1	1	0.000	0.000
Portugal	Aveiro	0	1	1	0.003	0.003
Portugal	Braganca	0	2	2	0.001	0.002
Portugal	Horta	1	1	3	0.282	0.846
Portugal	Lisbon	0	1	1	0.002	0.002
Portugal	Paco de Arcos	0	1	1	0.000	0.000
Portugal	Porto	0	1	1	0.000	0.000
Romania	Brasov	1	3	4	0.323	1.292
Romania	Bucharest	0	2	2	0.006	0.012
Romania	Piatra Neamt	0	1	1	0.166	0.166
Russia	(not set)	0	1	1	0.000	0.000
Russia	Moscow	0	2	2	0.000	0.000
Russia	Saint Petersburg	0	1	1	0.020	0.020
Russia	Volzhskiy	0	1	1	0.000	0.000
Senegal	Dakar	0	1	1	0.053	0.053
Singapore	(not set)	0	4	4	0.000	0.000
Singapore	Singapore	0	1	1	0.017	0.017
South Africa	Cape Town	3	7	11	0.029	0.319
South Africa	Centurion	0	1	1	0.000	0.000
South Africa	Mthatha	0	1	1	0.000	0.000
South Africa	Pretoria	1	0	1	0.000	0.000
South Africa	Stellenbosch	1	1	2	0.452	0.904
South Korea	Cheongwon-gun	0	1	2	0.000	0.000
South Korea	Dangjin-si	0	1	1	0.000	0.000
South Korea	Hwaseong-si	0	1	2	0.000	0.000
South Korea	Jeju-si	0	1	1	0.000	0.000
South Korea	Seoul	0	1	1	0.000	0.000
Spain	Barcelona	0	4	4	0.004	0.016
Spain	Granada	0	1	1	0.000	0.000
Spain	Palma	0	1	1	0.001	0.001
Spain	Sant Cugat del Valles	1	1	2	0.263	0.526
Spain	Valencia	14	5	38	3.581	136.078
Sweden	Orebro	0	1	1	0.000	0.000
Sweden	Solleftea	0	1	1	0.000	0.000
Sweden	Solna	1	6	8	0.293	2.344
Sweden	Stockholm	0	1	1	0.003	0.003
Sweden	Umea	0	1	1	0.008	0.008
Switzerland	Bern	0	1	1	0.000	0.000
Switzerland	Cham	0	1	1	0.011	0.011
Switzerland	Geneva	0	2	2	0.001	0.002
Switzerland	Zurich	0	1	1	0.000	0.000
Taiwan	(not set)	1	5	9	0.248	2.232
Taiwan	Dayuan District	0	1	1	0.000	0.000
Tanzania	Arusha	0	1	1	0.000	0.000
Thailand	Bangkok	0	1	1	0.000	0.000
Thailand	Mueang Chachoengsao District	0	1	1	0.011	0.011
Turkey	Ankara	2	1	3	0.598	1.794
Turkey	Canakkale	0	1	1	0.008	0.008
Turkey	Istanbul	1	2	3	0.007	0.021
Uganda	Entebbe	2	0	3	0.176	0.528
Ukraine	Kharkiv	0	1	1	0.002	0.002
Ukraine	Kropyvnytskyi	1	0	1	0.000	0.000
Ukraine	Kyiv	5	1	6	0.073	0.438
United Arab Emirates	(not set)	0	5	5	0.000	0.000
United Arab Emirates	Abu Dhabi	0	1	1	0.000	0.000

Country	City	Users	New_Users	Sessions	Avg Session Duration (hr)	Time of Use (hr)
Bolivia	(not set)	0	1	1	0.000	0.000
Bosnia & Herzegovina	(not set)	0	1	1	0.000	0.000
Côte d’Ivoire	(not set)	0	2	2	0.370	0.740
Ecuador	(not set)	0	2	2	0.059	0.118
Hong Kong	(not set)	2	7	11	0.662	7.282
Iran	(not set)	0	2	2	0.004	0.008
Jordan	(not set)	0	1	1	0.004	0.004
Lebanon	(not set)	1	1	2	0.000	0.000
Oman	(not set)	0	1	1	0.002	0.002
Peru	(not set)	3	14	20	0.063	1.260
Poland	(not set)	0	1	1	0.130	0.130
Qatar	(not set)	0	1	1	0.091	0.091
