# Supplementary material for: MicrobeTrace: Retooling molecular epidemiology for rapid public health response
Source: PLoS Comput Biol. 2021 Sep 7;17(9):e1009300. doi: 10.1371/journal.pcbi.1009300 (PMC8491948; doi:10.1371/journal.pcbi.1009300)
Supplement: S2 Table — (DOCX) [file pcbi.1009300.s004.docx]

| Input | Length (base pairs) | Count (taxa) | Compute time (seconds) | Layout time (seconds) | Time to figure (seconds) |
| --- | --- | --- | --- | --- | --- |
| HIV *pol* | 1,300 | 250 | 0.5 | 2 | 2.5 |
| HIV *pol* | 1,300 | 500 | 1 | 3 | 4 |
| HIV *pol* | 1,300 | 750 | 2 | 4 | 6 |
| HIV *pol* | 1,300 | 1,000 | 4 | 7 | 11 |
| HIV *pol* | 1,300 | 1,500 | 10 | 8 | 18 |
| HIV *pol* | 1,300 | 2,000 | 20 | 10 | 30 |
| HIV *pol* | 1,300 | 2,500 | 28 | 15 | 43 |
| HIV *pol* | 1,300 | 3,500 | 55 | 22 | 77 |
| HIV *pol* | 1,300 | 5,000 | 124 | 35 | 159 |
