# Supplementary material for: MicrobeTrace: Retooling molecular epidemiology for rapid public health response
Source: PLoS Comput Biol. 2021 Sep 7;17(9):e1009300. doi: 10.1371/journal.pcbi.1009300 (PMC8491948; doi:10.1371/journal.pcbi.1009300)
Supplement: S3 Table — Dashes in the last row indicate that MicrobeTrace has reached the upper limit of processing, and is unable to compute a network. (DOCX) [file pcbi.1009300.s005.docx]

| Input | Length (base pairs) | Count (taxa) | Compute time (seconds) | Layout time (seconds) | Time to figure (seconds) |
| --- | --- | --- | --- | --- | --- |
| SARS-CoV-2 whole genome | 30,000 | 250 | 4 | 2 | 6 |
| SARS-CoV-2 whole genome | 30,000 | 500 | 12 | 15 | 27 |
| SARS-CoV-2 whole genome | 30,000 | 750 | 35 | 31 | 66 |
| SARS-CoV-2 whole genome | 30,000 | 1,000 | 52 | 42 | 94 |
| SARS-CoV-2 whole genome | 30,000 | 1,500 | 190 | -- | -- |
