# Supplementary material for: MicrobeTrace: Retooling molecular epidemiology for rapid public health response
Source: PLoS Comput Biol. 2021 Sep 7;17(9):e1009300. doi: 10.1371/journal.pcbi.1009300 (PMC8491948; doi:10.1371/journal.pcbi.1009300)
Supplement: S4 Table — (DOCX) [file pcbi.1009300.s006.docx]

| Input | Count (taxa) | Compute time (seconds) | Layout time (seconds) | Time to figure (seconds) |
| --- | --- | --- | --- | --- |
| Newick | 50 | 0.05 | 0.01 | 0.06 |
| Newick | 100 | 0.1 | 0.02 | 0.12 |
| Newick | 200 | 1.5 | 1 | 2.5 |
| Newick | 500 | 27 | 6 | 33 |
| Newick | 1,000 | 130 | 45 | 175 |
| Newick | 1,250 | 373 | 60 | 433 |
