# Supplementary material for: MicrobeTrace: Retooling molecular epidemiology for rapid public health response
Source: PLoS Comput Biol. 2021 Sep 7;17(9):e1009300. doi: 10.1371/journal.pcbi.1009300 (PMC8491948; doi:10.1371/journal.pcbi.1009300)
Supplement: Attachment — Submitted filename: ResponsesToReviewers_final.docx [file pcbi.1009300.s007.docx]

1) What is the scalability of MicrobeTrace on a standard computer (e.g., Dell XPS or MacBook Pro)? I would have appreciated a figure that showed computation time with increasing numbers of sequencing or network data. For example, how many aligned SARS-CoV-2 genomes can the stand-alone version handle? How big (taxa/nodes) of a phylogeny or network can be rendered? What happens when you add GPS data? This performance data is crucial for analyzing the utility to the end user.

**Thank you for the feedback, we have responded with both a figure and a table that describe MicrobeTrace’s performance under various operating conditions. These include (a) partial polymerase *(pol)* HIV-1 sequence sets ranging from 250-5000 taxa, (b) whole genome SARS-CoV-2 sequence sets, ranging from 250-1000 taxa, (c) generic Newick trees, ranging from 100-1250 taxa. Adding GPS data does not impact computational times. We have also added text describing this analysis (Line 184)**

2) One of the most common questions that public health investigators ask the laboratorians (or the bioinformatician that is performing the genomic analysis) relates to inferred transmission events among cases. How closely related are the pathogen genomes? Does this mean that they are related in a transmission event? MicrobeTrace currently offers methods to set thresholds when viewing networks or phylogenies, but what can be done in terms of providing the end user a probability of transmission based on all of the epi and molecular/genomic data they are co-visualizing. Can algorithms be put in place to at least provide some guidance on the relatedness of cases? A number of researchers are working in this area (e.g., Xavier Didelot’s TransPhylo that uses epi and genomic data to assign probabilities for transmission events or Christoph Fraser’s PhyloScanner).

**Again, we appreciate the feedback and understand the critical importance of relating these genomic data back to the relevancy of transmission events. Given the complexity surrounding transmission inference, and its variability between pathogens and with respect to time, make it an intractable problem in a web browser application. That being said, we do offer the capability to integrate output from an external tool to augment a node or genetic link. Toward that end, we too have used these methods with tools like PhyloScanner to evaluate transmission networks.**

3) Throughout the development of MicrobeTrace, were end-users (county and state level epi staff on the investigations side) engaged to identify what data, reports, or visualizations are useful to them? If so, what was the feedback? If not, why and are there plans on doing so? I feel this is essential for ensuring widespread adoption and implementation. Perhaps canned reports could be made available based on the data types added?

**We appreciate this feedback and are delighted to address it in the paragraph below. In the intervening months since submission, we have developed a view specific to Hantavirus (called** [**HantaNet**](https://cdcgov.github.io/HantaNet/)**) that is our first attempt at tailoring MicrobeTrace for canned reports for specific pathogens. Given that this is an active area of development not known to the public we feel that it may be premature to comment in this area:**

***End users of HIV-TRACE were integral in the development of the minimum viable product and initial offering of MicrobeTrace in June 2018. From that point forward, end users at federal, state, and local levels were critically important in the development, testing, and addition of all features. Engagement with end users occurs on a continuous basis through multiple modalities, from email and instant messaging to screenshare sessions, in-person seminars, and webinars. The most valuable feedback in refining MicrobeTrace has been derived from screenshare sessions with end users at state and local levels who are engaging with the tool during an active outbreak investigation.***

4) Are the future plans to integrate MicrobeTrace with electronic laboratory and case reporting systems? This has been a huge bottleneck at the state health department level as many ELR and ECR systems are still poorly integrated. Now, as states add genomic data, there is no easy way to link it to case data. If would be great if MicrobeTrace could fill this need.

**We have begun evaluating data collection from a variety of sources, from cloud datastores to local implementations of REDCap, EpiInfo, or MS Access. This is likely to take the form of a Docker application that is configured to be executed and configured behind the user’s institutional firewall. While we understand the importance of filling this need, it is premature for us to discuss in this setting because it is an active area of development for our team.**
